# Zinc oxide nanoparticles induce oxidative stress, genotoxicity, and apoptosis in the hemocytes of *Bombyx mori* larvae

**DOI:** 10.1038/s41598-023-30444-y

**Published:** 2023-03-02

**Authors:** Rania Belal, Abir Gad

**Affiliations:** 1 Department of Genetics, Faculty of Agriculture, University of Alexandria, Alexandria, 21545 Egypt; 2 Department of Applied Entomology and Zoology, Faculty of Agriculture, University of Alexandria, Alexandria, 21545 Egypt

**Keywords:** Genetics, Zoology

## Abstract

The expanded uses of zinc oxide nanoparticles (ZnO-NPs) have grown rapidly in the field of nanotechnology. Thus, the increased production of nanoparticles (NPs) increases the potential risks to the environment and occupationally exposed humans. Hence, safety and toxicity assessment including genotoxicity of these NPs is indispensable. In the present study, we have evaluated the genotoxic effect of ZnO-NPs on 5th larval instar of *Bombyx mori* after feeding on mulberry leaves treated with ZnO-NPs at concentrations 50 and 100 μg/ml. Moreover, we evaluated its effects on total and different hemocyte count, antioxidant potential and catalase activity on the hemolymph of treated larvae. Results showed that ZnO-NPs at concentrations of 50 and 100 µg/ml have significantly decreased the total hemocyte count (THC) and different hemocyte count (DHC) except the number of oenocytes as they were significantly increased. Gene expression profile also showed up-regulation of *GST, CNDP2* and *CE* genes suggesting increase in antioxidant activity and alteration in cell viability as well as cell signaling.

## Introduction

Due to their unique physical and chemical properties, nanoparticles (NPs) are widely used in various production fields such as biomedicine, electronics, agriculture, cosmetics, food packaging, energy applications, and materials^1^. The increasing exposure to nanoparticles derives a great concern for human health and the environment. Recently, several studies showed that silver oxide (AgO), titanium dioxide (TiO_2_), and zinc oxide (ZnO) nanoparticles cause cytotoxicity, genotoxicity, apoptosis, oxidative stress, and inflammation^2–4^.

Zinc oxide (ZnO-NPs) is one of the most important metal oxide nanoparticles with many significant features, such as chemical and physical stability, high catalytic activity, effective antibacterial activity, as well as intensive ultraviolet (UV) and infrared (IR) adsorption^5^. However, recent studies reported the undesired toxic effects of ZnO NPs, as their application showed a decrease in cell viability, cellular damage, and cytotoxicity of MRC5 cells in human lungs and *Drosophila melanogaster *treated with ZnO-NPs^6^. Other toxic responses, including apoptosis and cytoskeleton changes, were demonstrated as toxic effects of ZnO-NPs in human neuroblastoma cells in a size-dependent manner^7^. In vitro studies revealed that the possible mode of action of ZNO-NPs toxicity is increased production of reactive oxygen species (ROS) followed by ROS-induction oxidative stress^8^. In addition to its great industrial importance, the silkworm, *Bombyx mori*, is considered an ideal research model in various fields such as bacterial pathogenicity, pharmacokinetics, and diabetes^9,10^ The purpose of this study is to look into the toxicological effects of ZnO-NPs on the hemolymph of *Bombyx mori* worms in order to assess the cytotoxicity and gene expression profile of larvae fed on Zinc Oxide.

## Materials and methods

### Chemicals

DPPH (1,1-diphenyl-2-picrylhydrazyl) and ZnO nanoparticles (ZnO-NPs) were purchased from Sigma–Aldrich, (Dorset, UK). All of the chemicals used were of the highest purity available from commercial sources.

### Insect strains

The larvae of *Bombyx mori* (Strain: Jingsong × Haoyue) were maintained in our laboratory and reared on mulberry (*Morus alba*) leaves under a 12-h light/12-h dark cycle. The larvae were fed three times per day.

### Experimental plan

The study was designed to examine the effect of ZnO-NPs on the 5th instar larvae of *B. mori* because silk protein synthesis occurs in this stage. ZnO-NPs (1.0 g) was mixed with 100 ml of distilled water, the mixture was treated by Ultrasonication for 20 min and then centrifuged for separation of solid and liquid phases. For bioassays, approximately ten mulberry leaves of the same size were dipped in 50 ml of each concentration of ZnO-NPs (50, 100, 500, and 1000 μg/ml) as a meal. Then, the leaves were left to dry for 10 min. After 48 h of the 5th instar larvae of silkworm, they were fed on treated leaves, while control larvae were fed on mulberry leaves dipped in distilled water three feedings/ day until the pre-pupal stage. The use of plant parts in the present study complies with institutional guidelines. Mortality of all concentrations groups and control was recorded daily.

### Sample collection

The hemolymph samples were collected from treated larvae with concentrations of 50 and 100 μg/ml in the pre-pupal stage to determine the antioxidant and Catalase activity. The samples were collected by creating a slit within the proleg. Hemolymph that flowed from the wound without hand pressure was collected into Eppendorf tubes containing 0.025% of phenylthiourea as an anti-melanization. The mean number of circulating hemocytes per mm^3^ was calculated by the formula of Jones^11^. To examine the differential hemocyte count (DHC), one hundred cells were identified to their typical hemocyte type after staining a smear of hemolymph with Wright's stain^2^.

### DPPH free radical scavenging activity

Relatively stable organic radical DPPH has been widely used in the determination of the antioxidant activity of hemolymph following the method of Brand William^12^. The solution of DPPH in methanol (6 × 10^−5^ M) was prepared just before ultraviolet radiation measurements. The hemolymph samples were added separately to the DPPH solution in a 1:1 ratio followed by a vortex. The reaction was taking place in the dark at room temperature under a nitrogen atmosphere. The absorbance was measured using an Ultraviolet–Visible spectrophotometer (Shimadzu UV-1601, Tokyo, Japan) at 517 nm was measured at different time intervals. The DPPH (containing no sample) was used as a control. The decreasing intensity of the purple color was taken as an increasing scavenging activity. Ascorbic acid served as a standard. The inhibition percentage of radical scavenging activity was calculated using the following equation.

$${\text{Inhibition}}\left( \% \right) = \left[ {\left( {{\text{Ac}} - {\text{As}}} \right)/{\text{Ac}}} \right] \times {1}00.$$

where Ac: Absorbance of control, As Absorbance of the sample.

### Catalase activity

CAT (EC 1.11.1.6) activity in hemolymph was assayed according to the method mentioned in^13^. In this method, a certain amount of phosphate buffer and hydrogen peroxide (H_2_O_2_) were added to hemolysate and analyzed for 3 min at 240 nm. Absorbance values were detected with an ultraviolet–visible spectrophotometer (Shimadzu UV-1601, Tokyo, Japan). Specific CAT activity was determined as the amount of decomposition of 1 mmol of H_2_O_2_ to water and oxygen per min per mg protein using the extinction coefficient value (e240 = 0.0394 mM^−1^ cm^−1^).

### RNA extraction and gene expression analysis

Total RNA was isolated from *Bombyx mori* larvae using Bio Basic RNA Extraction kit (Canada) according to the manufacturer’s instructions. RNA samples were obtained from the 5^th^ instar larvae fed on 100 ug/ml ZnO-NPs_._ 2 ugs of total RNA were subjected to reverse transcription reaction at 42 °C for 1 h using *Moloney Murine Leukemia* Virus (M-MLv) reverse transcription enzyme (Enzymotic Korea). The resulting c-DNA was amplified with primers specific for Arginine Kinase, Glutathione S-Transferase, Cytosolic Non-Specific Dipeptidase 2, and Calexcitine-2-like genes^14^. Gene names and primer sequences are shown in (Table 1). Quantitative real-time polymerase chain reaction (qRT-PCR) analysis was performed in the Rotor-Gene Q series (Qiagen) with the SYBR Green real-time PCR master mix (Enzymotic Korea) to monitor double-stranded DNA products. The data was analyzed using Rotor-Gene Q Series Software 2.0.3, and relative amounts of mRNA were calculated for the relatively expressed genes and the housekeeping gene *α-tubulin* using the comparative threshold cycle method. All samples were measured independently three times.

**Table 1:** Quantitative PCR primers sequences.

Gene name	Primer sequence from 5′ to 3′	
α-Tubulin	F: CTCCCTCCTCCATACCCT	R: ATCAACTACCAGCCACCC
Arginine kinase (*AK*)	F: ACGGTTGTTCAAGTGCCAGA	R:AGGAGGGTGGATCCGAATGA
Glutathion S- transferase 1 (*GST*1)	F: GGAAAGCTGACATGGGGTGA	R: AAGCCTTCACTTTGGGCTGT
Cytosolic nonspecific dipeptidase (CNDP2)	F: GCTCCACTCACTGAAACCGA	R: GGAACCACCGTTTTTGCTCC
Calexcitine- 2 like gene (CE)	F:GTCCATCGACAGCGAGGAAT	R: GGGCGTTCACATCCTCAGAA

### Nanoparticle characterization

The morphology of the nanoparticles was confirmed by TEM using an H-7500 transmission electron microscope (Hitachi, Japan) with an acceleration voltage of 80 kV. The nanoparticles were imaged after 10 μl of the NP suspension was air-dried on a carbon-coated 200-mesh copper grid. Suspensions containing nanoparticles were analyzed using a JEOL-TEM 100 CX instrument at the Electron Microscopic Unit, Faculty of Science, University of Alexandria. The obtained TEM micrographs were used to determine the shape and size of the nanoparticles^15^.

### Statistical analysis

All data results are expressed as the mean ± standard error. Experimental data were subjected to variance analysis using (ANOVA) test, and differences between means were tested for significance at 0.05 using an unpaired two-tailed T-test. For the statistical analysis of qRT-PCR, results are expressed as the mean, and error bars represent the standard deviation (+ S.D.). Differences between means were tested for significance at 0.05 using an unpaired, two-tailed *student test* and can be found in supplementary table S1.

## Results

### Characterization of ZnO-NPs

**Figure 1:**
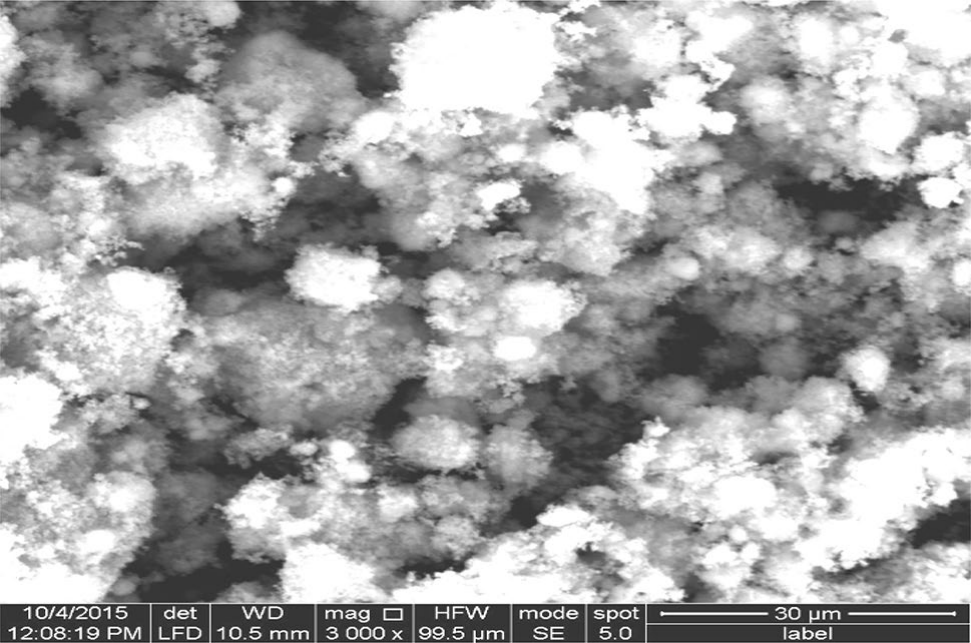
Transmission Electron Microscope (TEM) image of Zn-ONPs.

The ZnO-NPs used in our study exhibited spherical characteristics with absorbance spectra at λ max 425 nm. TEM images also substantiated the spherical zinc oxide nanoparticles with an approximate size of about 10 nm Fig. 1.

### Effect of feeding different concentrations of ZnO-NPs on survival rate of silkworm larvae

We raised silkworm 5th instar larvae in the lab with a normal diet of fresh mulberry leaves for control group or mulberry leaves supplemented with ZnO-NPs for treatment group. The growth status of the larvae was observed first, and then the survival rate of the larvae was obtained for each group. The larvae looked to be normal activity with both the control and treatments.

The tested concentrations were chosen due to their lowest mortality and negative impact of ZnO-NPs when compared with other highest concentrations (500 and 1000 μg/ml). The survival rate of the larvae was recorded to be 100% for the control group, 85% for concentrations 50 µg/ml, 66% for concentrations 100 µg/ml, and 34% and 20% for concentrations 500 and 1000 µg/ml, respectively.

### Effect of ZnO-NPs on total and differential hemocyte count in the hemolymph of *B. mori*

**Figure 2:**
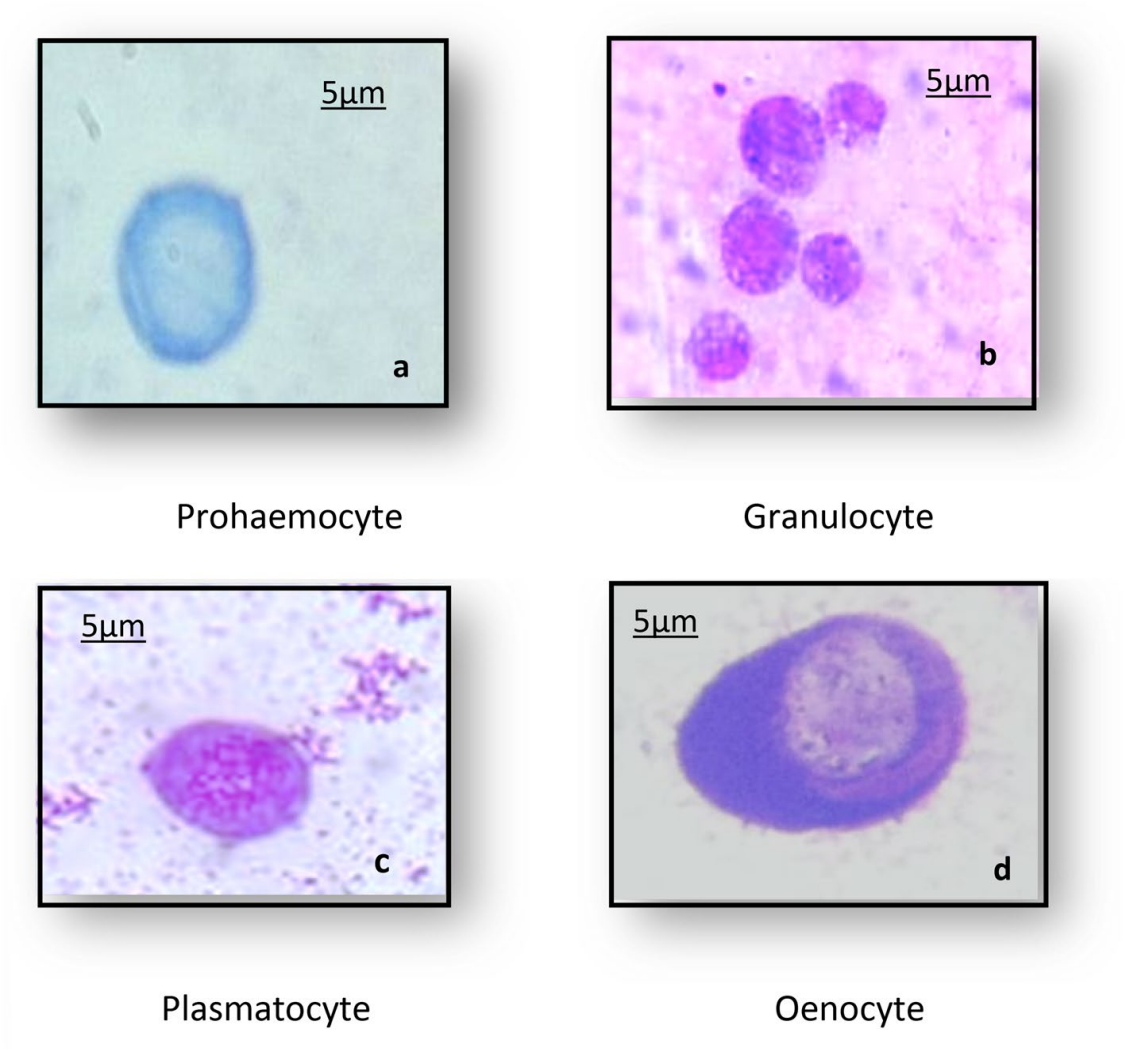
Hemocyte types of *Bombyx mori* larvae; (**a**) Prohemocyte (Prs) (**b**) Granulocyte (Grs) (**c**) Plasmatocyte(Pls) (**d**) Oenocytoid(Oes) (X100- oil; bar 5 μm)^2^.

In insect immunity, circulating hemocytes have crucial roles in both cellular mechanisms and producing antimicrobial components. Four basic types of hemocytes have been identified as Prohemocytes (Prs), Plasmatocytes (Pls), Granulocytes (Grs), and Oenocytes (Oes) Fig. 2.

The effect of ZnO-NPs on the total and differential hemocyte count of *B. mori* larvae was investigated and results revealed that the total hemocyte count (THC) significantly decreased in larvae exposed to 50 and 100 μg/ml of ZnO-NPs to be 2040 and 1950 cell/mm^3^ compared with the control group. (2706 cell/mm^3^). Furthermore, a significant decrease in the number of prohemocytes, granulocyte, and plasmatocyte was observed in larvae exposed to 100 μg/ml of ZnO-NPs about − 36.9, − 14.56 and − 12.14%, respectively, compared with the control group. Whereas the number of oenocytes significantly increased by about 89.2% (Table 2).

**Table 2:** Effects of ZnO-NPs on the total and different hemocyte count in 5th larval instar of *B. mori.*

Concentration (μg/ml)	THC mm3	Pr	Gr	Pl	Oe	Abnormal cells	Apoptotic cells
50	2040 ± 143^b^ (− 24.61%)	3.3 ± 0.4^b^ (− 28.26%)	40.5 ± 1.6^b^ (− 11.85%)	30 ± 1.7^a^ (− 6.54%)	10.8 ± 0.5^a^ (66.1%)	19	10
100	1950 ± 112^b^ (− 27.93%)	2.9 ± 0.2^c^ (− 36.9%)	38.7 ± 1.9^b^ (− 14.56%)	28.2 ± 1.7^b^ (− 12.14%)	12.3 ± 0.3^a^ (89.2%)	28	17
Control^2^	2706 ± 121^a^	4.6 ± 0.3^a^	45.3 ± 1.6^a^	32.1 ± 1.3^a^	6.5 ± 0.4^b^	0	0

Each value presents the mean ± SE.

*THC* total hemocyte count, *Pr* prohaemocyte, *Gr* granulocyte, *Pl* plasmatocyte, *Oe* oenocyte.

Means at each Colum followed by the same letter are not significantly different at 0.01.

Values in parentheses indicate the percentage decreased under the control.

### Abnormalities in the hemocytes after the application of ZnO-NPs

**Figure 3:**
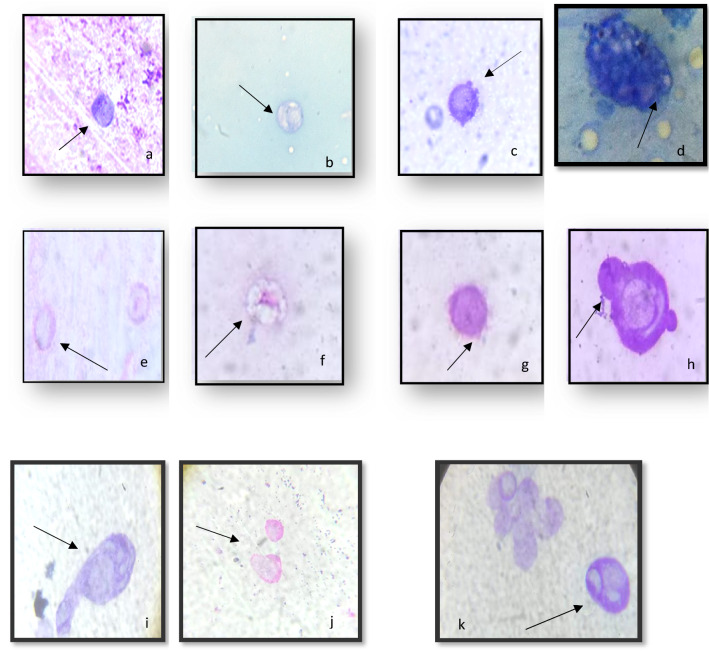
Effects of exposure to ZnO-NPs at concentrations 50 & 100 μg/ml on hemocyte morphology: (**a**) Reducing in cytoplasm, (**b**) Vacuolization in granulocyte, (**c**) Destroyed cell membrane (**d**) Numerous vacuoles covering the cytoplasm, (**e**) Nuclear degeneration, (**f**) Vacuolization in granulacyte (**g**) Protruded cytoplasmic contents (**h**,**i**), while at concentration100 μg/ml Cytoplasm lysis (**j**) Apoptotic cells (**k**) Mitotic division (X100- oil; bar 5 μm).

As illustrated in Fig. 3, three treatments with ZnO-NPs at concentrations of 50 and 100 g/ml resulted in varying degrees of deformation in all hemocyte types. Treatments with each of these concentrations resulted in Gr and Oe irregularities, reduced cytoplasm, and destroyed cell membranes (Plates a and b). Nuclear degeneration, cytoplasmic lysis, and the appearance of numerous cytoplasmic vacuoles were also observed. (Plates e, f, and d are examples.) In addition, ZnO-NPs treatments led to cell membrane rupture, mitotic division, cytoplasmic lysis, and protruded cytoplasmic contents. (Plates i,k,j,h). Concerning PLs, ZnO-NPs at both concentrations caused morphological deformities such as cytoplasm reduction, loss of pseudopods, and some cells losing their smooth cell boundary to become irregular (Plates c, g).

### Antioxidant assays

#### Free radical scavenging activity of DPPH

**Figure 4:**
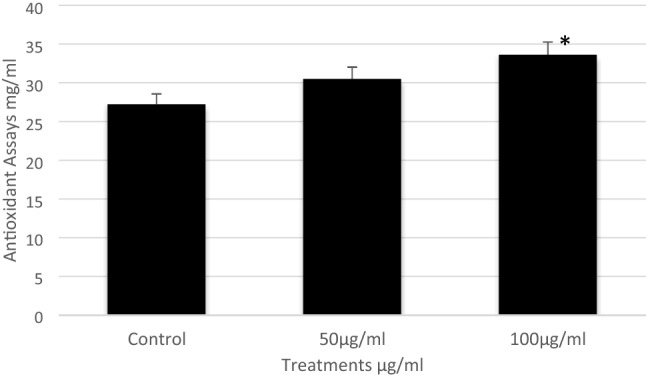
The inhibitory concentrations of ZnO-NPs on the 5th instar larvae of *B. mori*. Data are mean ± SE. Statistical analyses were performed using unpaired two-tailed Student's t-test (*p* < 0.05).

The DPPH scavenging assay exhibited the effective inhibitory activity of ZnO-NPs compared to the standard ascorbic acid. When ZnO-NPs were added to the DPPH solution, the color change, these change in color due to the scavenging of DPPH through a donation of the hydrogen atom to a stable DPPH molecule which was responsible for the absorbance at 517 nm^12^. The antioxidant activity of hemolymph from insects fed on ZnO-NPs significantly increased at concentrations (50 and 100 μg/ml) to 29.7 and 33.5 μg/ml, respectively while the control was 27.21 μg/ml (Fig. 4).

#### Antioxidant enzyme activities

**Figure 5:**
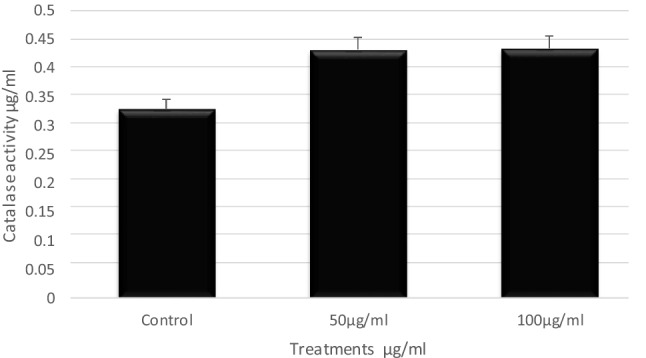
The antioxidant enzyme activities of ZnO-NPs on the 5th instar larvae of *B. mori.* Data are mean ± SE. Statistical analyses were performed using unpaired two-tailed Student's t-test (*p* < 0.05).

Results relating to catalase activity in the hemolymph of the 5th instar larvae, after being treated with different concentrations of ZnO-NPs are given in Fig. 5. Catalase activity in the hemolymph of the control group was detected as 0.33 mmol/min/mg protein, while it was significantly increased at concentrations of 50 and 100 μg/ml to be 0.432 and 0.456 mmol/min/mg protein, respectively.

### qRT-PCR of the differentially expressed genes

**Figure 6:**
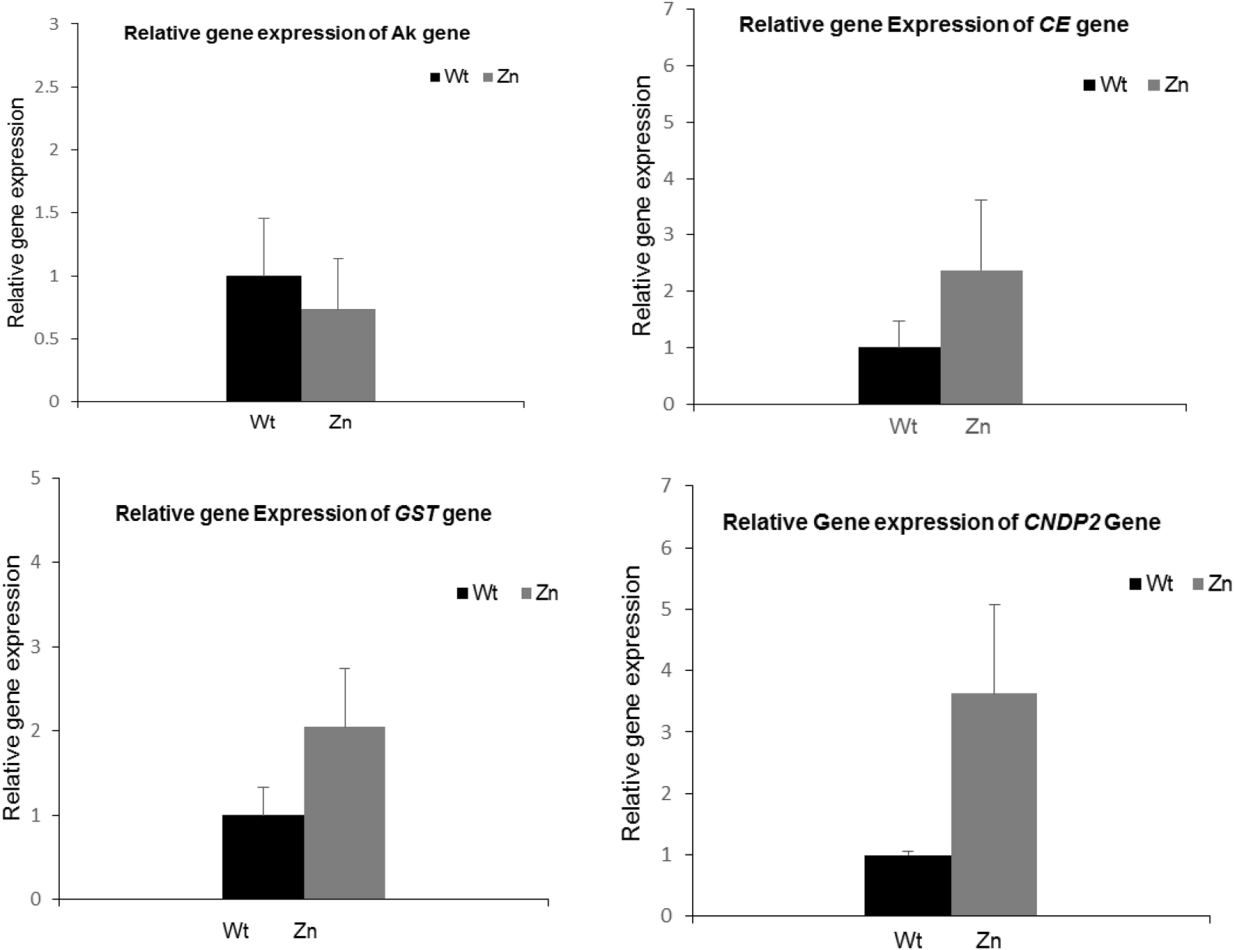
Quantitative RT-PCR of *Ak, GST, CNDP2* and CE genes in the 5th instar larvae of *B. mori* fed on 100 μg/ml ZnO-NPs. For each sample, RNA was extracted, transcribed to C-DNA and normalized to the house keeping gene *Tubulin α*. Error bar represents + SD.

The molecular approach was successfully used in recent studies to monitor gene expression differentiation under oxidative stresses in insects,^6,16,17^. Gene expression of genes known for their role in metabolism, insect immunity, cell signaling, and detoxification were analyzed in this study. Arginine Kinase (*AK*) is a primary enzyme in cell metabolism and adenosine 5′-triphosphate (ATP)-consuming processes. It plays an important role in cellular energy metabolism and maintaining constant ATP levels in invertebrate cells, in addition to its role in the silkworm immune response against viral infection^18,19^. Glutathione S-transferase *(GST)* contributes to the detoxification of both endogenous and xenobiotic compounds, and *GSTs* genes are involved in intracellular transport, biosynthesis of hormones, and protection against oxidative stress^20^. The cytosolic non-specific dipeptidase *2(CNDP2) gene* encodes a non-specific carnosinase, which is involved in the biosynthesis of *GSH* that acts as a detoxification agent in the body^21^. Calexcitine- 2-like genes *(CE)* are signaling proteins that bind calcium and GTP, inhibit potassium channels, and are involved in binding metal ions^22^. The expression analysis of these genes was processed by qRT-PCR reactions after exposure to ZnO-NPs. Compared to the control, the *AK* gene was slightly down-regulated while the *CE*, *GST*, and *CNDP2* genes were significantly up-regulated in the treated larvae Fig. 6.

## Discussion

Zinc oxide nanoparticles are widely used in biomedical productions due to their unique characteristics. Several studies, on the other hand, have found that ZnO-NPs can cause cytotoxicity, apoptosis,^23^, immune suppression^24^, cell cycle alteration, and DNA damage^25^. In this study, ZnO-NPs are administered via food, which is supposed to be one of the natural routes through which animals may be exposed to commonly used engineered NPs.

Our results showed that feeding the 5th instar larvae of *B. mori* on mulberry leaves treated with ZnO-NPs at concentrations of 50 and 100 μg/ml significantly affected the total and differential hemocyte counts of the silkworm larvae. The findings also showed that the toxic effect of ZnO-NPs on hemocytes caused dramatic irregularities in hemocytes, a reduction in the cytoplasm, and vacuolization of all cells, particularly granulocytes. The morphological changes of *B. mori* hemocytes were similar to the results that were previously obtained by^26^.They found that ZnO-NPs exposure caused some morphological changes in the cell membrane, cytoplasm, and nucleus. Also, treatment by ZnO-NPs has significantly increased the antioxidant potential of the hemolymph and the catalase activity. Similar results were observed by^2^ when studying the effect of AgO-NPs on the total and different hemocyte counts and the antioxidant activity in the hemolymph of treated *B. mori* larvae. Our results indicate an induction of oxidative stress in silkworms fed on ZnO-NPs.

In brief, these studies showed that ZnO-NPs caused adverse effects such as cytotoxicity, inflammation, and oxidative stress. Undoubtedly, ZnO-NPs were effective on *B. mori* at lower concentrations. This might be a response of the organism against the toxicity of ZnO-NPs and may also be related to the activity of the antioxidant enzymes. The antioxidant activity in the hemolymph of treated larvae was likely elevated by the initial increase in H_2_O_2_, then later began to decline with the activity of CAT, especially at concentrations of 100 μg/ml of ZnO-NPs, because CAT is an important antioxidant enzyme that responds to scavenge H_2_O_2_ concentrations,^27^. It is well known that CAT is found in nearly all living organisms and catalyzes the separation of H_2_O_2_ from water and molecular oxygen^28^. The most striking effect observed here was a tremendous increase in CAT activity when the last instars of *B. mori* were treated with 100 μg/ml of ZnO-NPs. This activity of CAT might be related to an adaptive response of the larvae to an increase in H_2_O_2_, because CAT regulates H_2_O_2_ concentration in living organisms during oxidative stress conditions,^14^. In general, antioxidant enzyme activities, particularly CAT activity, can be used to assess NP-induced oxidative stress in insects.

*Bombyx mori* was taken as a model organism for eukaryotes to study the effect of ZnO-NPs exposure to hemolymph as well as the expression of genes related to metabolism, detoxification, and cell signaling. Arginine kinase is an important enzyme in cellular energy metabolism as well as in the consumption of ATP^18^. Recently, it was detected that the *AK* gene plays an important role in the insect immune response towards nucleopolyhedrovirus (BmNPV)^19^. In this study, the relative gene expression analyses of the *Ak* gene revealed that it was insignificantly down-regulated, and this may be due to the decrease in the hemolymph surface of the treated larvae, resulting in low ATP needs. The *GST* gene is known for its contribution to detoxification against xenobiotics in addition to its major role in protecting against oxidative damage and antioxidant processes^29^. In the present work, the *GST* gene was significantly up-regulated after silkworms were fed on ZnO-NPs-treated leaves, consistent with the previous studies^14^, indicating the increase of *GST* gene expression in *B. mori* fed on Ag-NPs. These findings may be related to the glutathione S-transferase gene's role in detoxification against ROS activities. Cytosolic non-specific dipeptidase 2*(CNDP2)* (also named tissue carnosinase), belongs to the family of M20 metallopeptidases. It breaks down carnosine (-alanyl-L-histidine), a bioactive dipeptide^30^* CNDP-2*.The majority of recent researches were focused on human health. Up-regulation of the *CNDP 2* gene was detected in colon cancer cell lines^31^. However, research on *Drosophila melanogaster* revealed that the *CNDP-2* gene is located in the nucleus, where it can be associated with chromatin, implying that it plays a role in DNA replication, transcription, or repair^32^. In this work, *CNDP2* was significantly up-regulated after the exposure of larvae to ZnO-NPs, suggesting an alteration in the cell cycle growth of the worm. The Calexcitin (CE) gene, a low molecular weight signaling protein that binds to Ca^2+^ and GTP and regulates K1 channels^33^, was studied here. The CE gene was discovered to be specifically expressed in neuron cells in *Drosophila melanogaste*^34^. Our results showed a significant up-regulation of the *CE* gene in larvae fed with ZnO-NPs, suggesting changes in cell Ca^+^ signaling pathways correspond with those described in reference^25^, suggesting that ZnO-NPs decrease the viability of hemocytes.

## Supplementary Information

Supplementary Information.

## Data Availability

All data generated or analyzed are included in this manuscript and its supplementary data.
