# Supplementary material for: Zinc oxide nanoparticles induce oxidative stress, genotoxicity, and apoptosis in the hemocytes of Bombyx mori larvae
Source: Sci Rep. 2023 Mar 2;13:3520. doi: 10.1038/s41598-023-30444-y (PMC9981692; doi:10.1038/s41598-023-30444-y)
Supplement: Supplementary file 1 — Supplementary Information. [file 41598_2023_30444_MOESM1_ESM.pdf]

Zinc oxide nanoparticles induce oxidative stress, genotoxicity and apoptosis in the hemocytes of *Bombyx mori* larvae

Rania Belal[1] and Abir Gad[2]*

[1] Department of Genetics, Faculty of Agriculture, University of Alexandria, Alexandria 21545, Egypt. E-mail: rania.belal@alexu.edu.eg

[2]Department of Applied Entomology and Zoology, Faculty of Agriculture, University of Alexandria, Alexandria 21545, E-mail: abeer.gad@alexu.edu.eg

*Corresponding author: Abir Gad. Department of Applied Entomology and Zoology, Faculty of Agriculture, University of Alexandria, Alexandria 21545, E-mail: abeer.gad@alexu.edu.eg

S1: https://doi.org/10.1111/phen.12333

Toxicity effect of Silver Nanoparticles to the Haemocytes and Antioxidant activity of Silkworm Bombyx mori. Physiol Entomol (2020) 45:154–160.

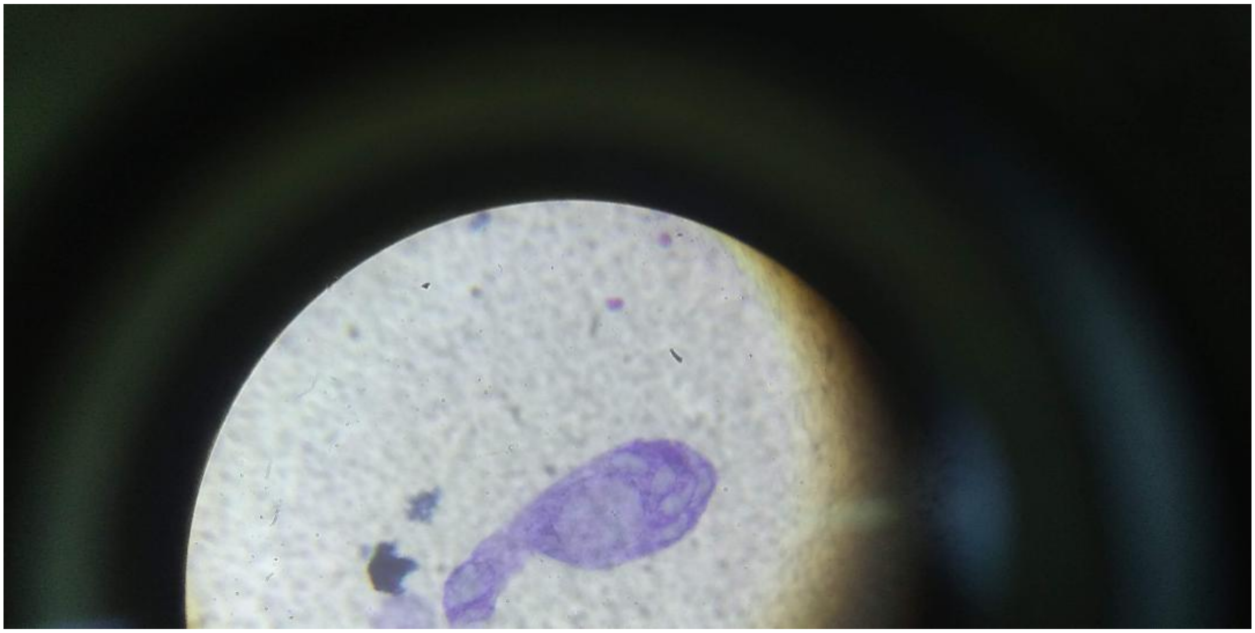

S2: Effect of ZNO- NPS at concentration 100 μg/ml on Oenocyte, the treatment caused cytoplasm lysis

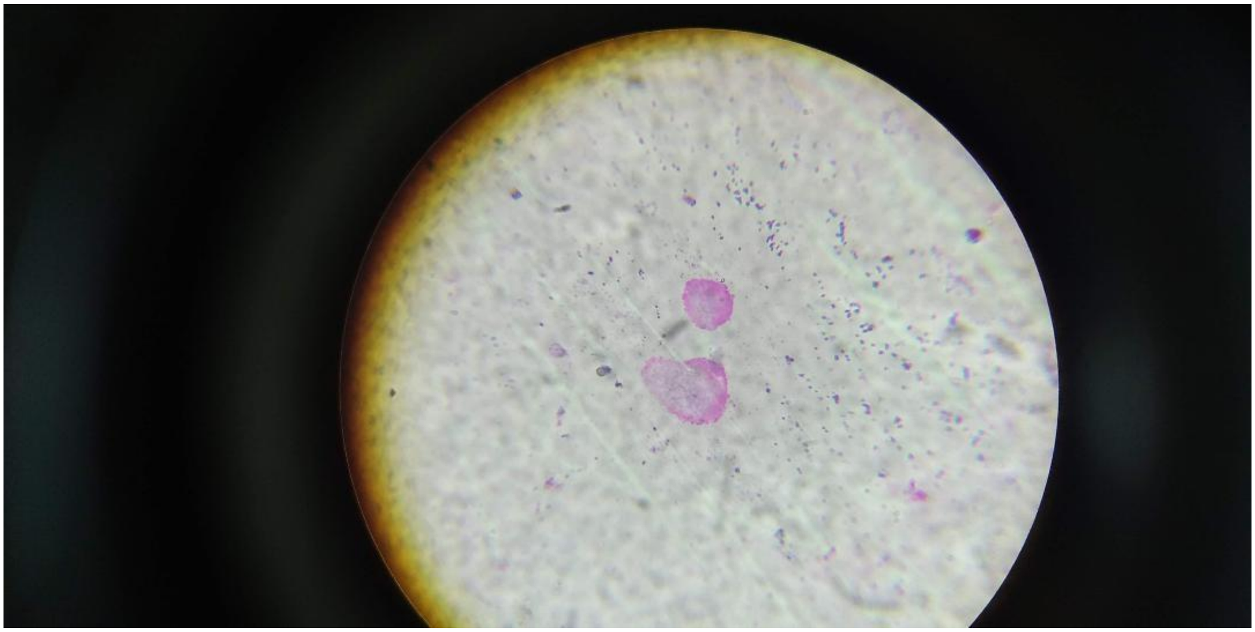

S3: Effect of ZNO- NPS at concentration 50 and 100 μg/ml on granulocyte, the treatment caused apoptotic cells

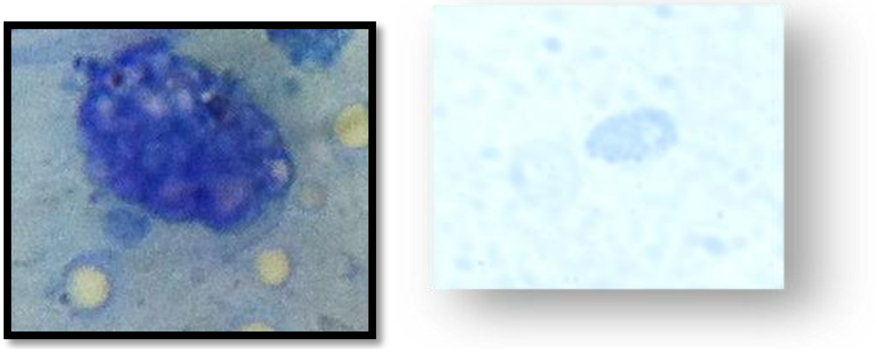

S4: Effect of ZNO- NPS at concentration 50 and 100 μg/ml on Oenocyte, the treatment caused numerous vacuoles covering the cytoplasm.

Gene Name [a]	Fold change [b]	Expression [c]	Student-test *P* value[d]
Arginine Kinase (*AK*)	0.735433	↓	0.6083
Glutathion S- transferase *(GST*	2.049114	↑	0.008556
Cytosolic non-specific dipeptidase *2(CNDP2)*	3.63	↑	0.005099
Calexcitine- 2 like genes *(CE)*	2.375	↑	0.004792

S5: Statistical analysis of real-time expression of studied genes.

[a] Gene name . [b] Fold change control/ treatment . [c] Gene expression :   describes the down regulation while ↑ describes the up regulation. [d] differences between means were tested for significance at 0.05 using T-test.
